# Antimicrobial and Anti-Biofilm Effect of an Electrolyzed Superoxidized Solution at Neutral-pH against *Helicobacter pylori*

**DOI:** 10.1155/2019/6154867

**Published:** 2019-12-18

**Authors:** Daniela Guadalupe Lucio-Sauceda, Víctor Hugo Urrutia-Baca, Ricardo Gomez-Flores, Myriam Angélica De La Garza-Ramos, Patricia Tamez-Guerra, Alonso Orozco-Flores

**Affiliations:** ^1^Laboratory of Immunology and Virology, School of Biological Sciences, Autonomous University of Nuevo Leon, Pedro de Alba and Manuel L. Barragán St., Cd. Universitaria, San Nicolás de los Garza, N.L., Monterrey, 66450, Mexico; ^2^Integral Dentistry Unit and Specialties, Center for Research and Development in Health Sciences, Autonomous University of Nuevo Leon, Dr. Aguirre Pequeño and Silao Ave., Mitras Centro, N.L., Monterrey, 64460, Mexico

## Abstract

The presence of *Helicobacter pylori* in the oral cavity has been associated to the failure of antimicrobial therapy in patients with gastrointestinal infection and the development of oral diseases. However, it has been reported that the maintenance of good oral hygiene can improve the therapeutic success rates, where the use of mouthwashes with anti-*Helicobacter* activity would help to achieve it. The aim was to evaluate the antimicrobial activity of OxOral® mouthwash against *H. pylori* and its effect on biofilm formation. The minimum inhibitory concentration (MIC) of OxOral® (pH = 6.4–7.5, ORP = 650–900 mV) against *H. pylori* was calculated testing serial dilutions 0.117–15 ppm against 1 × 10^8^ CFU/mL of *H. pylori* (ATCC® 700824™) by broth microdilution method using 96‐well plates. The *H. pylori* biofilm formation was determined by the optical density measurement at 600 nm from coverslips stained with 0.1% crystal violet. The gene expression of *ureA*, *luxS*, *flaA*, *omp18, * and *lpxD* were analyzed by RT‐qPCR. OxOral® cytotoxicity was evaluated in a human gingival fibroblast cell line by MTT assay. MIC was of 3.75 ppm, with 99.7 ± 7.7% bacterial growth inhibition. In the negative control, the biofilm formation was observed, whereas when bacteria were treated with OxOral® at 0.234, 0.469, and 0.938 ppm, an inhibition of 35.5 ± 0.9%, 89.1 ± 1.2%, and 99.9 ± 5.5% were obtained, respectively. The gene expression analysis showed that *flaA*, *omp18,* and *lpxD* genes were down‐regulated with OxOral® compared with control (*p* < 0.05). Low cytotoxicity of 16.5 ± 7.6% was observed at the highest dose (15 ppm); no significant differences were observed from 15 to 0.469 ppm compared to the control of untreated cells (*p* > 0.05). Our results reveal an important anti-*Helicobacter* activity of OxOral® and open the possibility of its therapeutic use new studies, which would increase the success rate of conventional therapies against *H. pylori*.

## 1. Introduction

The *Helicobacter pylori* gastric infection has been associated with the development of chronic gastritis, peptic ulcer, and gastric cancer [1, 2]. Thus the Agency for Research on Cancer (IARC) recognized *H. pylori* as a group 1 carcinogen to humans [3, 4]. *H. pylori* infection varies between 50.8% in developing countries compared to 34.7% in developed countries [5]. For the treatment of infected patients, a combination of two antibiotics plus a proton pump inhibitor (PPI) called triple therapy is recommended. However, an increase in antimicrobial has been reported, which varies in different geographical areas. In Latin America, 12% antimicrobial primary resistance clarithromycin, 53% for metronidazole, 4% for amoxicillin, 6% for tetracycline, 3% for furazolidone, 15% for fluoroquinolones, and 8% for dual clarithromycin and metronidazole have been found [6, 7]. In Italy, significantly higher of resistance rates have been reported, 25% resistance to clarithromycin [8].

Some bacterial pathogens are able to form biofilms as an important virulence factor to overcome environmental stress and the drugs, as *Staphylococcus epidermidis* attaching to various surfaces of medical devices [9]. The *H. pylori* persistence in human infections and its antimicrobial resistance in conventional therapy have been attributed not only to genetic variability, but also to ability of *H. pylori* to form biofilm. Several reports indicate that *H. pylori* forms biofilm either *in vitro* or *in vivo* [10, 11].

On the other hand, studies have shown that fecal-oral transmission of *H. pylori* is the main route of infection [12]. In recent years, several studies have suggested the oral cavity would play an essential role as an extra-gastric reservoir in the oral-oral transmission of the infection; however, this is controversial [13, 14].

It is known that the presence of *H. pylori* in the oral cavity is one of the leading causes of the reappearance of gastric infection and that the treatment of oral infection significantly increases the eradication of *H. pylori* infection in the stomach. Miyabayashi et al. [15], observed that patients with oral *H. pylori* had a significantly higher risk of gastric reinfection after receiving adequate antimicrobial treatment. Therefore, adequate oral hygiene could be an alternative to increase the rates of therapeutic success, where the use of mouthwashes could help in their maintenance [16].

Recently, novel oral antiseptics based on electrolyzed water (EW) with antimicrobial activity against oral pathogens have been developed [17–20]. EW is the product of the electrolysis of a diluted solution of NaCl in an electrolysis cell, inside which a diaphragm (partition or membrane) separates the anode and the cathode [21].

Electrolyzed waters have been used as an alternative in the field of asepsis and sanitization for several years. Initially, its application on human tissues was avoided due to its physical–chemical characteristics such as very acid or alkaline pH, instability, and toxicity. However, its use was implemented in agriculture, the food industry, and in the disinfection of various surfaces and inert materials by its high microbicidal capacity [22]. Thus, electrolyzed solutions of superoxidation (ESS) with a near‐neutral pH (6.4–7.5) have been obtained by ionic selectivity, which, in addition to having high microbicidal activity and stability, are safer [23–26]. Currently, ESS has become a new alternative for tissue asepsis in different areas of medicine such as dentistry, surgery, dermatology, treatment of burns, and diabetics. In the literature, studies have been described that show that the application of ESS, for example, in dental, surgical, burnt, and diabetic patients, significantly reduces the tendency to infections, bleeding time and pain; it also accelerates tissue regeneration [27–31]. However, the effectiveness of oral antiseptics based on ESS against *H. pylori* is unknown.

Therefore, this study focuses on the evaluation of the antimicrobial and antibiofilm activity of a novel ESS with neutral pH, called OxOral® against *H. pylori* by in vitro assays.

## 2. Materials and Methods

### 2.1. Bacterial Culture Conditions

*Helicobacter pylori* (ATCC® 700824™) was obtained from the American Type Culture Collection (ATCC, Rockville, MD). Activation of *H. pylori* was performed in 5 mL of trypticase soy broth (TSB; Becton Dickinson, Franklin Lakes, NJ), supplemented with 5% (v/v) heat inactivated fetal bovine serum (FBS; Gibco, Gaithersburg, MD). After, 50 *μ*L of bacterial suspension was plated on trypticase soy agar (TSA; Becton Dickinson), supplemented with 5% (v/v) defibrinated sheep blood (DSB), as reported by Urrutia-Baca et al. [32]. Broth and agar cultures were placed into a sealed jar with CampyGen sachet (Oxoid Ltd, Basingstoke, UK) to maintain microaerobic conditions and incubated at 37°C for seven days.

### 2.2. Electrolyzed Superoxidized Solution (ESS) at Neutral pH Properties

The ESS at neutral pH, called OxOral® mouthwash antiseptic, was elaborated and supplied by Esteripharma (Mexico city, Mexico). OxOral® has a pH from 6.4–7.5 with a concentration of active species (mainly as HOCl/ClO^–^, O_3_, H_2_O_2_, ClO_2_, and Cl_2_) of 15 ppm (0.0015%) and an oxide-reduction potential (ORP) from 650 to 900 mV at room temperature.

### 2.3. Antibacterial Activity Assay

The minimal inhibitory concentration (MIC) was performed in 96-well flat-bottom plates containing 100 *μ*L of each OxOral® dilution in TSB supplemented with 10% FBS and 100 *μ*L of 1.0 × 10^8^ CFU/mL of *H. pylori*, up to a final volume of 200 *μ*L per well; 5 *μ*g/mL tetracycline was used as a positive control for inhibition of bacterial growth and saline solution (SS) as a negative control. The plates were incubated at 37°C for 72 h in a microaerobic atmosphere; microbial growth was indicated as a change in optical density at 600 nm. Subsequently, the percentage of growth inhibition was calculated using

(1) $$\begin{array}{c} \%\text{inhibition}=100-\left\{\left[\frac{\left(\text{Sample}-\text{Positivecontrol}\right)}{\left(\text{Negativecontrol}-\text{Positivecontrol}\right)}\right]\times 100\right\} \end{array}$$

the MIC value was defined as the lowest concentration of OxOral® that inhibited 99% of *H. pylori* growth. Finally, 10 *µ*L of culture were taken from wells without *H. pylori* growth and spread on TSA supplemented with 5% DSB. The CFU were counted after seven days of incubation. The minimum bactericidal concentration (MBC) value was established as the lowest concentration of OxOral® that killed *H. pylori* (without CFU).

### 2.4. H. pylori Biofilm Formation Assay

The ability of *H. pylori* to form biofilm on abiotic surfaces was carried by total bacterial count using sterile 22 × 22 mm glass coverslips were placed in 100 × 15 mm Petri dishes. Each plate was filled with 12 mL of MH broth supplemented with 0.3% glucose and 10% FBS and 2 mL of OxOral® until reaching a final concentration from 0.938 ppm to 0.117 ppm; 5 *μ*g/mL tetracycline and SS were used as positive and negative controls, respectively. Biofilm formation was started by inoculating *H. pylori* at an initial concentration of 1 × 10^6^ CFU/mL. The dishes were incubated under microaerobic conditions at 37°C for 168 h without agitation. After incubation, coverslips were washed three times with 1X phosphate buffered saline (PBS) to remove planktonic cells and biofilm residues. The samples were dried and stained with 0.1% violet crystal (CV) for 30 s. After staining, the coverslips were rinsed with distilled water to remove excess dye and air-dried at room temperature for 30 min. For the biofilm quantification, the dye associated with the biofilms was dissolved using 33% glacial acetic acid and optical density was measured at 600 nm using a microplate reader; the percentage of biofilm inhibition was calculated.

### 2.5. The Gene Expression Assay

OxOral®-treated cells at 0.938 ppm coming from biofilm assay were detached from the coverslip surface by vortexing for 10 min in 20 mL of 1X PBS. The collected bacteria were centrifuged at 4,000 × g for 10 minutes, then washed and resuspended in 100 *μ*l of 1X Tris-EDTA buffer (TE; pH = 7.4) containing 5 mg/mL of lysozyme (Sigma-Aldrich, St. Louis, MO) and 10 mg/mL of Proteinase K (Thermo-Fisher, Carlsbad, CA, USA). The mixture was incubated at 37°C for 30 min and the total RNA was extracted using Trizol (TRI Reagent; Sigma-Aldrich) according to the manufacturer's protocol. For the synthesis of complementary DNA, M-MLV Reverse Transcriptase kit (Promega, Madison, WI) was used following the instructions.

For quantitative PCR (qPCR), reported and validated primers for *lpxD*, *omp18*, *ureA*, *flaA,* and *luxS* gene; 16 s rRNA were used as reference gene (Table 1).

Each qPCR was prepared using 12.5 *μ*L of 2X Maxima Sybr Green/qPCR master mix (Thermo-Scientific, Carlsbad, CA, USA), 0.5 *μ*M of forward/reverse primer mixture, 100 ng of cDNA and nuclease-free water up to a final volume of 25 *μ*L were mixed in 96-well plates. The qPCR was run using a LightCycler 480II thermal cycler (Roche, Basel, Switzerland) with a four-step program: one cycle of pre-incubation, 50 cycles of amplification, one cycle for melting curve, and one cooling cycle. The pre-incubation was at 95°C for 10 min and ramp rate of 4°C/s, each amplification cycle was carried out in three steps: the denaturing at 95°C for 10 s and ramp rate of 4°C/s, the annealing at temperature according to each pair primer for 15 s and ramp rate of 2°C/s, and the extension to 72°C for 10 s and ramp rate of 4°C/s in individual acquisition mode. The melting curve was at 95°C for 5 s 4°C/s, 65°C for 1 min 2.2°C/s, and 97°C in continuous acquisition mode at 5°C. The cooling was at 40°C for 30 s with a ramp rate of 1.5°C/s. For gene expression analysis, the cycle threshold (CT) values and the normalized relative expression ratio were calculated by the ΔΔCT method using LightCycler 480II software (Roche, Basel, Switzerland) and Rest2009 (QIAGEN, Hilden, Germany).

### 2.6. Cytotoxicity Assay

The cytotoxic effect of OxOral® against a human gingival fibroblast cell line (ATCC®PCS-201-018™) was evaluated by 3-(4, 5-dimethylthiazol-2-yl)-2, 5-diphenyl tetrazolium (MTT) assay. The cell line was cultured in Dulbecco's modified Eagle's medium (DMEM; Gibco), supplemented with 10% FBS (Sigma-Aldrich), 1X antibiotic-antimycotic (Gibco), and 6 mM L-glutamine (Gibco) called complete DMEM at 37°C for 48 h, in a humidified atmosphere of 5% CO_2_. One hundred microliters of complete DMEM containing 5 × 10^4^ cells was placed into each well of flat-bottom 96-well plate (Corning Inc, Corning, NY) and grown to approximately 90% confluence. After, 100 *µ*L of OxOral® dilution (from 15 to 0.469 ppm) were added to each well and incubated for 24 h; DMEM and 5% triton X-100 were used as negative and positive control, respectively. After incubation, supernatant was discarded, cells were carefully washed with PBS, and 100 *μ*L of 0.5 mg/mL MTT (Sigma-Aldrich) in DMEM were added to the wells. The plates were incubated for 4 h, then supernatant was discarded and 200 *μ*L of dimethyl sulfoxide (Sigma-Aldrich) were used to dissolve formazan crystals. Optical density was read at 570 nm using a microplate reader. The percentage of cytotoxicity was calculated using $\%\mathrm{cytotoxicity}=100-\left\{\left[\frac{\left(\text{Sample}-\text{Positivecontrol}\right)}{\left(\text{Negative}\mathrm{control}-\text{Positivecontrol}\right)}\right]\times 100\right\}.$

### 2.7. Statistical Analysis

The results were expressed as mean ± standard deviation (SD) of the response of three replicate determinations per treatment, from three independent experiments. The level of significance was assessed by ANOVA, post-hoc Tukey HSD, Student's t, and Dunnett's tests (*P* < 0.05), using IBMSPSS statistics software v22.

## 3. Results

### 3.1. Antimicrobial Effect of OxOral® against H. pylori

**Figure 1:**
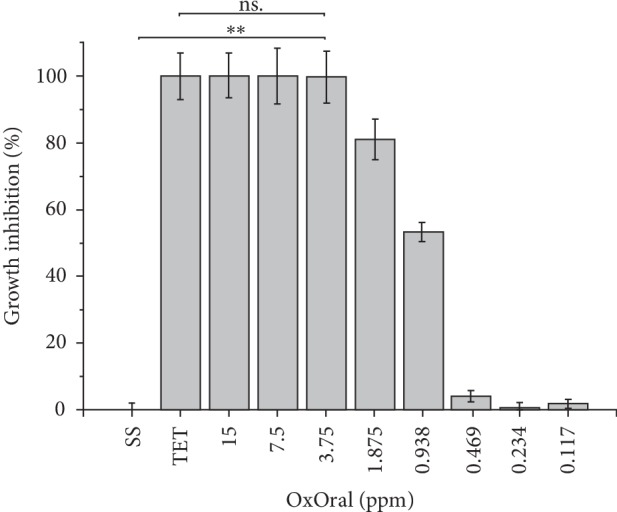
Effect of OxOral® on *H. pylori* growth. 1 × 10^8^ CFU/mL of *H. pylori* (ATCC® 700824™) suspension was treated with 15–0.117 ppm OxOral® in 96-well microtiter plates. Plates were then incubated for 72 h in a microaerobic atmosphere at 37°C. After incubation, the effect of OxOral® on *H. pylori* growth was determined by measuring optical densities at 600 nm and the percentage of growth inhibition was calculated, as explained in the text. The data represent the percentage mean ± the percentage deviation. TET, 5 *μ*g/mL tetracycline; SS, saline solution; ∗∗, *p* < 0.001; ns, not significant.

An important antimicrobial activity of OxOral® was observed from 15 to 3.75 ppm, in those doses no difference was observed compared to 5 *μ*g/mL tetracycline (*p* > 0.05). Therefore, 3.75 ppm of OxOral® with 99.7 ± 7.7% inhibition was established as the MIC value. In addition, a residual inhibitory effect was observed at 1.875 ppm and 0.938 ppm with 81.1 ± 6.0% and 53.3 ± 2.8%, respectively (Figure 1). The MBC value was 7.5 ppm when no visible growth was observed.

### 3.2. Anti-Biofilm Effect of OxOral® against H. pylori

**Figure 2:**
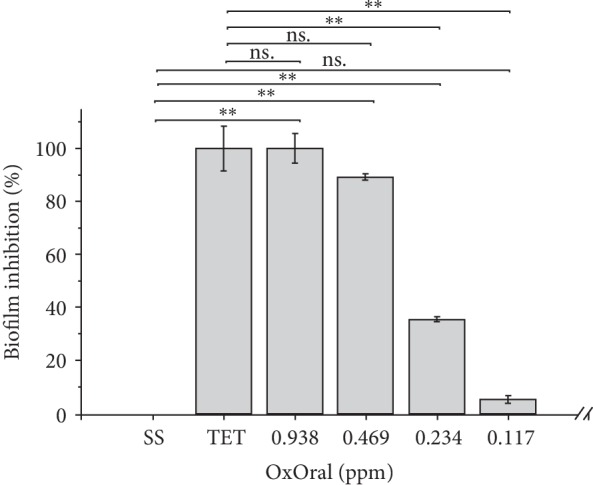
Evaluation of the *H. pylori* biofilm formation in the presence of OxOral®. The *H. pylori* biofilm formation was carried using sterile 22 × 22 mm glass coverslips that were placed in Petri dishes. Each plate was filled with MH broth supplemented and OxOral^®^ at different doses. Biofilm formation was started by inoculating *H. pylori* at an initial concentration of 1 × 10^6^ CFU/mL. The dishes were incubated under microaerobic conditions at 37°C for 168 h. After incubation, coverslips were washed and stained with 0.1% violet crystal. After staining, the dye associated to biofilms was dissolved using 33% glacial acetic acid, then the optical density was measured at 600 nm and the percentage of biofilm inhibition was calculated. The data represent the percentage mean ± the percentage deviation. TET, 5 *μ*g/mL tetracycline; SS, saline solution; ∗∗, *p* < 0.001; ns, not significant.

**Figure 3:**
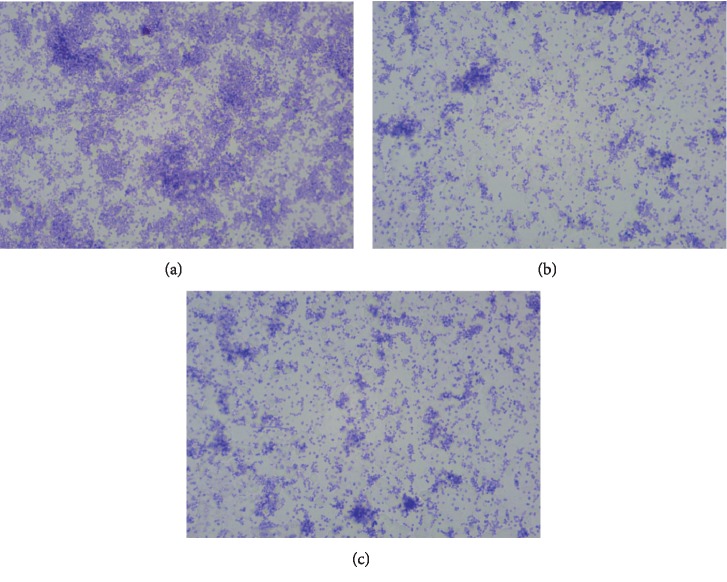
The *H. pylori* biofilm treated with OxOral®. The *H. pylori* biofilm formation was carried using sterile glass coverslips that were placed in Petri dishes. Each plate was filled with MH broth supplemented and OxOral® at different doses: (a) saline solution, (b) 0.938 ppm OxOral**®**, and (c) 0.469 ppm OxOral**®**. Biofilm formation was started by inoculating *H. pylori* at an initial concentration of 1 × 10^6^ CFU/mL. The dishes were incubated under microaerobic conditions at 37°C for 168 h. After incubation, coverslips were washed and stained with 0.1% violet crystal. The coverslips were observed by optical microscopy at a magnification of 100x.

Our results showed no inhibition of *H. pylori* biofilm in the presence of SS. However, when biofilm cells were treated with 0.938 ppm, 0.469 ppm, and 0.234 ppm of OxOral® values of 99.9 ± 5.5%, 89.1 ± 1.2%, and 35.5 ± 0.9% and of inhibition were obtained, respectively (Figure 2); 5 *μ*g/mL tetracycline showed an inhibition of 100 ± 13.5%. From a concentration of 0.117 OxOral® no difference was observed compared to SS control (*p* > 0.05). The *H. pylori* biofilm cells were observed using a microscope with a magnification of 100X (Figure 3).

### 3.3. Effect of OxOral® on the Expression of Genes Associated with the Formation of Biofilm in H. pylori

**Figure 4:**
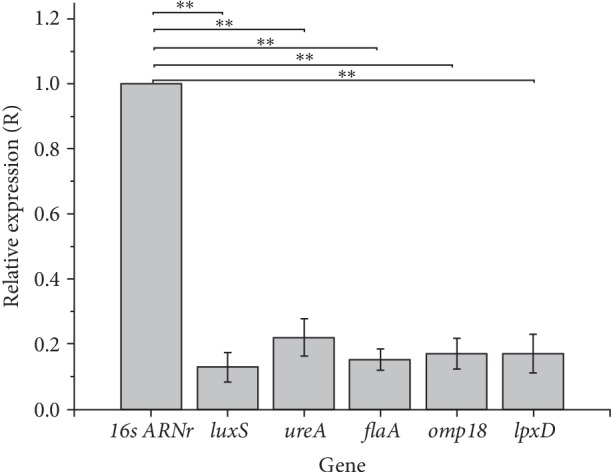
Effect of OxOral® on mRNA relative expression levels of *H. pylori* biofilm genes. The relative expression was determined by the ΔΔCT method and the relative expression ratio (*R*) was calculated. Data represent means ± SE of triplicate determinations from three independent experiments; 16s rRNA gene was used as reference. ∗∗, *p* < 0.001.

After evaluating the effect of OxOral® at 0.938 ppm on the relative expression of the genes associated with the adhesion and biofilm formation mechanisms in *H. pylori*, the results demonstrated that *flaA*, *omp18,* and *lpxD* genes were down regulated with a relative expression ratio (*R*) of 0.152 (SE = 0.120–0.197), 0.170 (SE = 0.123–0.267), and 0.171 (SE =0.113–0.254), respectively. The gene most affected by the OxOral® treatment was the *luxS* with an (*R* = 0.127) (SE = 0.082–0.216), while the least affected was the *ureA* gene with an (*R* = 0.220) (SE =0.162–0.353). Based on the statistical analysis, significant differences were observed between the samples treated with OxOral® and untreated control (*p* < 0.001), as shown in Figure 4.

### 3.4. Low Cytotoxicity of OxOral® on Human Gingival Fibroblast Cells

**Figure 5:**
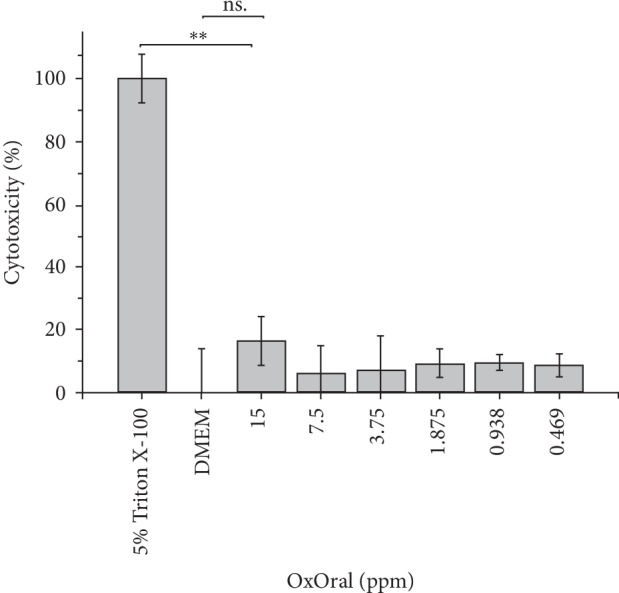
Evaluation of the OxOral® cytotoxicity on ATCC®PCS-201-018™ cell line. Human gingival fibroblast cells (5 × 10^4^ cells/mL) were incubated in the presence of OxOral® at various concentrations for 24 h. After incubation, the cytotoxicity of OxOral® on ATCC®PCS-201-018™ cell line was determined by the MTT reduction assay. The optical densities was measured at 570 nm and the percentage of cytotoxicity was calculated. The data represent the percentage mean ± the percentage deviation. ∗∗, *P* < 0.001; ns, not significant.

The greatest cytotoxic effect was at 15 ppm of OxOral® after 24 h of treatment with OD 590 nm = 0.20558 ± 0.01577 and 16.5 ± 7.6% of cytotoxicity. No significant differences were observed at concentrations from 0.469 ppm to 15 ppm of OxOral® compared to DMEM control (*p* > 0.05). The results are shown in Figure 5.

## 4. Discussion

The increase of multidrug resistant pathogenic bacteria is a major concern in the world for being one of the most common causes of morbidity and mortality. In the case of *H. pylori*, strains resistant to the first-line antibiotics used in the treatment of gastrointestinal infection have been reported, which has limited their success rate. In addition, many researchers have detected *H. pylori* in the oral cavity in both dental plaque and saliva [33]. Some studies have shown that the presence of oral *H. pylori* is related to the reappearance of gastric infection leading to the failure of the therapies, and that periodontal treatment and good oral hygiene in infected patients significantly increase the *H. pylori* eradication rate in the stomach [13, 34]. In addition, oral *H. pylori* infection has been associated to periodontitis and gingivitis [35]. Some reports that include clinical and experimental studies have reported that individuals with oral *H. pylori* infection tend to develop periodontal disease [36].

Disinfection is one of the most important factors to prevent or treat infections and oral pathologies. Currently, there are oral antiseptic or mouthwash formulations, based on compounds such as chlorhexidine, cetylpyridinium chloride, hexetidine, triclosan, and super-oxidized water. The selection of appropriate oral disinfectants that possess broad spectrum activity against different microorganisms including *H. pylori* could provide adequate oral hygiene and support conventional therapy against this gastrointestinal pathogen. In this study we evaluated the antimicrobial potential of OxOral® at 0.0015% (15 ppm) against *H. pylori*.

Some studies have reported the important antimicrobial activity of electrolyzed super-oxidized water. Lee and Choi [18], evaluated the antibacterial effect of puri-water on aerobic and anaerobic bacteria in saliva, a significantly inhibition of bacterial growth compared to tap water (*p* < 0.05) was observed.

There are different antimicrobial broad spectrum disinfectants and mouthwashes manufactured by Esteripharma®, Mexico, S.A. of C.V, each with different compositions and proposes. Velázquez-Meza et al. [37] evaluated the antimicrobial activity of a disinfectant based on super-oxidized water called Estericide Qx® against 524 bacterial clinical isolates causing nosocomial infections, including Gram-negative (*Escherichia coli* and *Pseudomonas aeruginosa* beta-lactam resistant) and Gram-positive (*Staphylococcus aureus* and *S. epidermidis* methicillin resistant, and *Enterococcus faecium*). The MIC assays showed that the isolates were inhibited at concentrations of 10–40 ppm. For Gram-positive bacteria, the MIC values 20 and 40 ppm were more predominant (95% of isolates), whereas for Gram-negative bacteria, the MIC values 10 and 20 ppm had the highest percentage (91.7% of isolates). The difference between the two groups was statistically significant (*p* < 0.001). The results showed that Estericide Qx® provides a broad spectrum antibacterial activity mainly in gram-negative.

Landa-Solis et al. [38], treated pure cultures of *S. aureus*, *E. coli*, *P. aeruginosa*, *Salmonella typhi*, and *Candida albicans* with Microcyn® and found it was active on all bacteria and C. albicans tested. Vorobjev et al. [39] reported that super oxidized solution was effective on spores, gram-positive, and gram-negative bacteria causing nosocomial infections. Gunaydin et al. [40], found good antimicrobial activity of Medilox® super oxidized solution in ATCC strains and clinical isolates.

In our study, we established the MIC = 3.75 ppm and MBC = 7.5 ppm values of OxOral® mouthwash against *H. pylori*, these concentrations are lower compared to the studies mentioned above for other microorganisms.

There are few reports about the anti-*Helicobacter* effect of ESS. Masuda et al. [41], reported significant bactericidal activity against *H. pylori* using ESS (ORP = 1,100 mV and pH = 2.5) for the disinfection of endoscopes. Shetty et al. [42], evaluated the microbicidal activity of Sterilox® at 144 ppm (ORP >950 mV and pH = 5.0–6.5) and against *Clostridium difficile* spores, *H. pylori*, vancomycin resistant *Enterococcus* species, *C. albicans* and several *Mycobacterium* species for the disinfection of endoscopy units. Sterilox® showed a microbicidal activity against *H. pylori* equal to other conventional methods of disinfection (2% glutaraldehyde and 0.35% peracetic acid). However, these studies did not evaluate different concentration of ESS; our study is the first to bring to light the MIC and MBC values of ESS against *H. pylori*.

According to many studies, the presence of chlorine and a high concentration of ORP in ESS seem to be the responsible of its antimicrobial activity. Active chlorine compounds can destroy the membranes of microorganisms, but other modes of chlorine action (e.g., decarboxylation of amino acids, reactions with nucleic acids, and unbalanced metabolism after the destruction of key enzymes) also have been proposed [22]. Studies suggest that HOCl is the most active of the chlorine compounds. HOCl penetrates cell membranes and produces hydroxyl radicals, which exert their antimicrobial activity through the oxidation of key metabolic systems. In addition, OH- hydroxyl radicals, which are the strongest oxidizing agents, also have shown antimicrobial activity [43].

Biofilms are communities of bacteria associated with the surface that are embedded in a hydrated matrix of extracellular polymeric substances. It is known that the formation of biofilms is one of the mechanisms of resistance to antimicrobial drugs and adverse environmental conditions that many bacterial pathogens can develop. It has recently been suggested that the formation of biofilms plays a role in the gastric colonization of *H. pylori*. However, the role of *H. pylori* biofilms in the failure of antimicrobial treatment and the survival of this pathogen has not been established. In addition, relatively little is known about the structure of the *H. pylori* biofilm or the genes associated with this mode of growth [44].

There are reports about the effect of electrolyzed super-oxidized solution with neutral pH on the formation of biofilms against some bacterial pathogens including *Listeria monocytogenes*, *E. coli*, *P. aeruginosa*, and *S. aureus* on steel, plastic, and glass surfaces as well as fruits and vegetables [45–48].

Zan et al. [49], investigated the antibacterial effects of super-oxidized water (SPO) on root canals infected with biofilms of *Enterococcus faecalis*. They used sodium hypochlorite (NaOCl), which did not show statistically significant differences compared to three and five minutes of irrigation with SPO (*p* > 0.05). However, NaOCl did show statistically significant differences among the other groups (Saline and Medilox®). They reported in terms of successful endodontic treatment that the super-oxidized water does have a remarkable bactericidal effect similar to that of traditional NaOCl against *E. faecalis* biofilms, which can be used as an effective irrigation solution.

In our study, we observed a significant reduction in the formation of *H. pylori* biofilms using OxOral® mouthwash at concentrations from 15 ppm to 0.234 ppm; there is no precedent direct report to our study. Some authors have evaluated products related, such as OxOral® sterilizing and OxOral® aseptic flush against biofilms of *E. faecalis*, they observed null anti-biofilm activity compared to 5.25% NaOCl [50, 51]. However, the results of our study would open the possibility of the therapeutic use of OxOral® mouthwash in *H. pylori*- infected patients for the purpose of reduce the *H. pylori* permanence in the oral cavity.

The evaluation of both *in vitro* and *in vivo* toxicity of products based on super-oxidized water is undoubtedly one of the crucial points for its implementation and use in humans. Aras et al., evaluated the toxicity of a super-oxidized water (SOW) in Wistar-albino rats who were administered intraperitoneally 10 mg/kg of SOW as a single and multiple dose (day 1, 3 and 5). All rats treated with SOW survived after administration. In addition, macroscopic and microscopic examinations revealed no pathological and toxicity findings in the peritoneal cavity and liver or signs of complications [52].

A similar study by same author evaluated the effect of SOW on the uterus and ovary when administered by intraperitoneal infusion in a rat model. All rats remained healthy after one week of follow-up. The macroscopic and microscopic examinations of the groups treated with SOW (single and multiple doses of SOW) did not show significant differences compared to the control group (saline solution). Microscopic examination revealed glandular structures in the uterus and functional follicles in different stages of maturation in the ovary, demonstrating that the intraperitoneal infusion of SOW does not produce any significant toxicity and complications in the tissues of uterus and ovary [53]. Our *in vitro* results suggest that OxOral® does not produce cytotoxicity to human gingival fibroblast cells after 24 h of treatment; the highest percentage of cytotoxicity was 16.5% in the highest dose (15 ppm).

The development of biofilm is achieved through a series of sequential steps marked by changes of gene expression in response to environmental signals and highly regulated cell-cell signaling, called quorum sensing (QS). The QS system, normally associated with the regulation of virulence factors, also regulates the various stages of biofilm development from the initial adhesion to the final detachment of the cells [54]. In *H. pylori*, the AI‐2 autoinducer encoded by the *luxS* gene plays an important role in the stimulation of the *lux* operon [55]. The expression of *luxS* is essential in the mechanisms of adhesion, motility, and represents a significant indicator of the production of biofilm in which bacteria migrate and adhere to microcolonies [56].

Apart from the *luxS* gene, other genes are involved in the formation of biofilm, including genes encoding flagella (*flaA*), pili type I and type IV and surface adhesins [44]. It has been reported that *flaA* is an indispensable factor in the mechanism of colonization, adhesion, and the formation of biofilm. Eaton et al., demonstrated that *flaA*-deficient mutant strains showed a complete loss of motility and a significant reduction in colonization of the gastric epithelium [57]. On the other hand, it has been reported that the expression of *flaA* depends on *luxS* and its transcription increases with the density of the culture [55].

The outer membrane proteins (OMP) are important for ion transport, osmotic stability, bacterial virulence, and adhesion. The *omp18* gene is a lipoprotein precursor associated with peptidoglycan, present in *H. pylori*, which is involved in adhesion to gastric cells [58]. The cell envelope gene (*lpxD*) encoding UDP-3-0-(3-hydroxymyistoyl) glucosamine N-acyltransferase is up-regulated after adhesion to gastric cells *in vitro* [59]. Therefore, genes *omp18* and *lpxD* could participate in the formation of biofilm.

The *ureA* virulence gene encodes the A subunit of the urease enzyme required for colonization and maintenance of the organism in hostile environments. The enzyme urease of *H. pylori* is essential for the neutralization of the stomach pH. The loss of urease activity acidifies the biofilm, decreasing the stability of the bacterial community [60].

Our results showed a decrease in the expression of the *luxS*, *lpxD*, *flaA*, *urea* and *omp18* genes of *H. pylori* in the presence of OxOral® that could suggest a potential mechanism of negative regulation of OxOral® on the *H. pylori* biofilm formation.

On the other hand, recent studies have shown that coinfections with *H. pylori* and other microorganisms (viruses) have been associated with the development of gastric diseases, as the role of *H. pylori* and the Epstein-Barr virus (EBV) in gastric carcinogenesis [61]. The elimination of oral and gastric *H. pylori* in EBV-infected patients could have a favorable impact on the prognosis of patients, specifically in the prevention of cancer.

The limitation of our study was that all the experiments were performed only on an ATCC strain of *H. pylori*.

## 5. Conclusions

Our *in vitro* results on antimicrobial and anti-biofilm effect of OxOral® mouthwash against *H. pylori*, and low cytotoxicity open the possibility of its therapeutic use in *H. pylori*-infected patients as adjuvant in conventional therapy.

## Figures and Tables

**Table 1:** Specifications of the primers used for RT-qPCR assays.

Gene	Forward 5′–3′	Reverse 5′–3′	Fragment length (bp)	Reference
*ureA*	CTG ATG GGA CCA AAC TCG TAA	TTG CCT TCG TTG ATA GTG ATG	109	In this study
*omp18*	TGC ACG ATC TCA TCT AAA GTC TC	CGG GAC TAT CAT CGC TTC TAT TT	87	
*lpxD*	GTT TAG GCT CAT TCA CGC TTT G	TCG TGG ATA ACC CGC ATT TAG	93	

*flaA*	CAG TAT AGA TGG TCG TGG GAT TG	GAG AGA AAG CCT TCC GTA GTT AG	127	Urrutia-Baca et al. [32]
*luxS*	CTA AAT TCT GTG CGC CCT CTA A	ACG ATG CAA GAC GTG CTA AA	100	
*16s* ARNr	GGA GTA CGG TCG CAA GAT TAA A	CTA GCG GAT TCT CTC AAT GTC AA	127	

## Data Availability

The data used to support the findings of this study are available from the corresponding author upon request.
